# Pure sensory Guillain-Barré syndrome: A case report and review of the literature

**DOI:** 10.3892/etm.2014.1955

**Published:** 2014-09-11

**Authors:** JINGJING YANG, MINGMING HUAN, HUAJUN JIANG, CHUNLI SONG, LIN ZHONG, ZHANHUA LIANG

**Affiliations:** 1 Department of Neurology, First Affiliated Hospital of Dalian Medical University, Dalian, Liaoning 116011, P.R. China; 2 Department of Orthopedics, First Affiliated Hospital of Dalian Medical University, Dalian, Liaoning 116011, P.R. China; 3 Department of Pathology, First Affiliated Hospital of Dalian Medical University, Dalian, Liaoning 116011, P.R. China

**Keywords:** Guillain-Barré syndrome, prednisone, intravenous immunoglobulin, sural nerve biopsy, demyelinating neuropathy

## Abstract

Sensory Guillain-Barré syndrome (GBS) is an acute demyelinating neuropathy that presents clinically with involvement of the sensory peripheral nerve only. To date, <10 cases of pure sensory GBS have been reported; thus, the clinical and pathological features of sensory variant GBS are yet to be well characterized. The current study reports the case of a 43-year-old female that presented with acute, symmetric and monophasic sensory neuropathy, without motor weakness. Patient history, clinical examination, routine nerve conduction studies and sural nerve biopsy were reviewed. All the observations were consistent with a diagnosis of pure sensory GBS. In particular, the pathological features of the sural nerve biopsy revealed that the form of regenerated nerve fibers have complete structure of myelinated nerve fascicles, and these myelinated nerve fibers are thicker than other parts of the biopsy. The patient received small-dose (20 mg/day) prednisone initially, but without any benefit. Satisfactory improvements were observed with one course of intravenous immunoglobulin.

## Introduction

Sensory Guillain-Barré syndrome (GBS) is an acute demyelinating neuropathy that presents clinically with involvement of the sensory peripheral nerve only. However, the existence of a purely sensory form of GBS remains subject to controversy, since these cases always demonstrate a degree of motor weakness or abnormalities in motor nerve conduction studies (NCSs) and are difficult to distinguish from acute sensory neuronopathy (1). To date, only a few cases of pure sensory GBS have been reported (2–4), with the majority of cases being anecdotal and few studies describing a peripheral nerve pathology. Thus, the clinical and pathological features of sensory variant GBS have not been well characterized, and reduced awareness of these features has resulted in delays in the diagnosis and treatment.

The current study reports the case of a 43-year-old female who presented with symptoms consistent with a diagnosis of pure sensory GBS. The patient exhibited satisfactory improvements following one course of intravenous immunoglobulin. Diagnosing sensory GBS is important since immunotherapy may positively influence the prognosis, in contrast to the slow but steady progression associated with idiopathic sensory neuropathy or paraneoplastic sensory neuronopathy. Therefore, understanding the pathological and clinical features may aid in the diagnosis of complicated clinical cases and prevent unnecessary procedures.

## Case report

A 43-year-old female developed numbness of the distal lower limbs that extended to all the limbs over four days, and was admitted to the First Affiliated Hospital of Dalian Medical University (Dalian, China) on April 26, 2011. The patient experienced nonspecific flu-like symptoms and suffered from a mild sore throat during the two weeks prior to admission. After a few days, the patient developed numbness on the soles of both feet, which progressed over two days to the knees; thus, the patient had difficulty with walking due to poor balance. Subsequently, the patient had a markedly unsteady gait and tingling sensations in the distal lower limbs, which increased in intensity and extended more widely. Clumsiness in the upper limbs and pseudoathetosis was observed occasionally. The time when the symptoms of the disease of the patient had reached their peak was achieved within three weeks.

**Figure 1:**
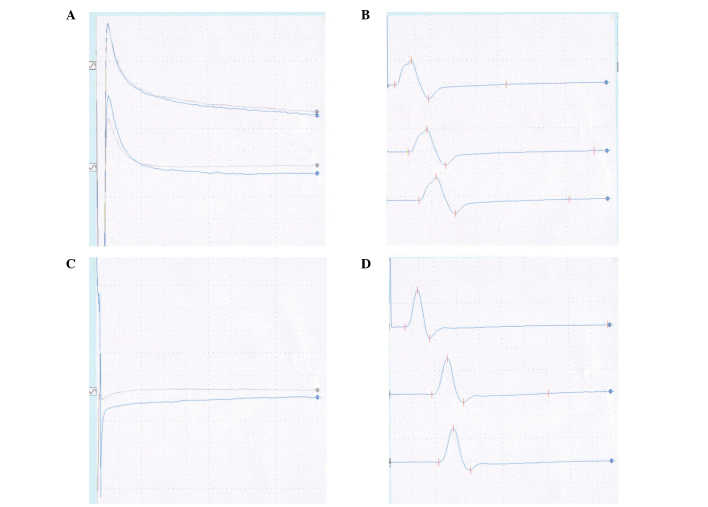
Results from the NCSs showing (A) sensory, left ulnar, absent sensory potentials, (B) motor, left ulnar, normal motor potentials, (C) sensory, right sural, absent sensory potentials and (D) motor, right peroneal, normal motor potentials. NCSs, nerve conduction studies.

**Figure 2:**
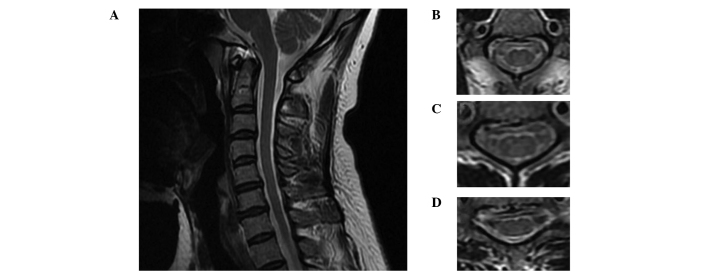
MRI scans of the spinal cord. (A) Sagittal T2-weighted MRI scan showing the normal appearance of the posterior column, despite a number of disc osteophyte complexes with mild central canal stenosis. Axial T2-weighted MRI scans at the (B) C2, (C) C4 and (D) C6 levels. MRI, magnetic resonance imaging.

**Figure 3:**
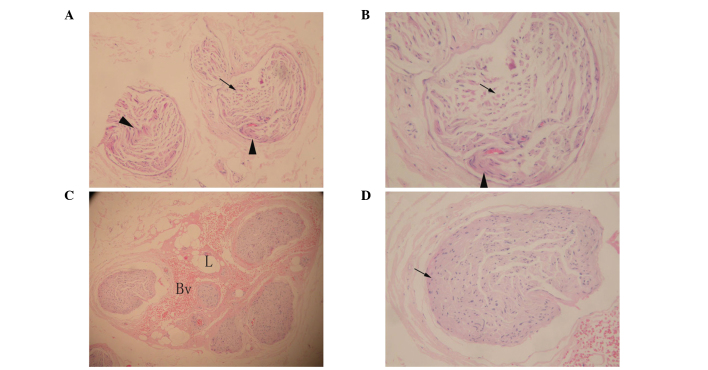
Hematoxylin and eosin stained images of the sural nerve. (A and B) Mild loss of myelinated fibers and a few thinly myelinated fibers (arrow) were observed, as well as regenerated nerve fibers (arrow head). (C and D) Normal controls (arrow head). A and C magnification, ×200; B and D magnification, ×400.

**Figure 4:**
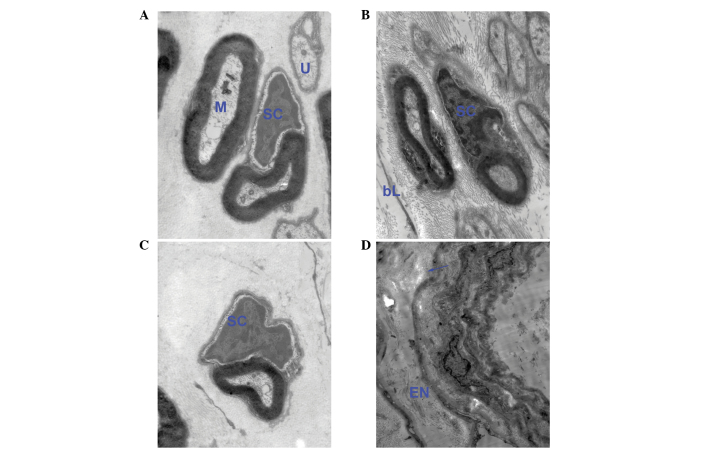
Electron microscopy of the sural nerve (magnification, ×5,000). (A–C) Transverse sections showing normal myelinated (M) and unmyelinated (U) axons, with Schwann cell (SC) nucleus breakdown. (D) Axon clusters surrounded by more than one endoneurial (EN) lamina (arrow).

Routine laboratory tests were conducted on the second day following admission. Cerebrospinal fluid (CSF) routing revealed 1,194 mg/l of protein and a cell count of 2×10^6^/l, while other issues were normal. Routine NCSs revealed absent sensory potentials, while the motor NCS was normal (Fig. 1). Sagittal T2-weighted magnetic resonance imaging (MRI) scans (Fig. 2) of the cervical spine revealed a normal appearance of the posterior column, despite a number of disc osteophyte complexes with mild central canal stenosis at the cervical level. Pathological observations of the sural nerve biopsy were conducted. Light microscopy (Fig. 3) showed moderate subperineurial edema, mild loss of myelinated fibers and a few thinly myelinated fibers without inflammatory changes. In particular, the form of regenerated nerve fibers have complete structure of myelinated nerve fascicles, and these myelinated nerve fibers are thicker than other parts of the biopsy. Electron microscopy (Fig. 4) revealed normal myelinated and unmyelinated axons, however, the Schwann cell nucleui were broken down and the cytoplasm of the Schwann cells were pale watery moderately.

**Figure 5:**
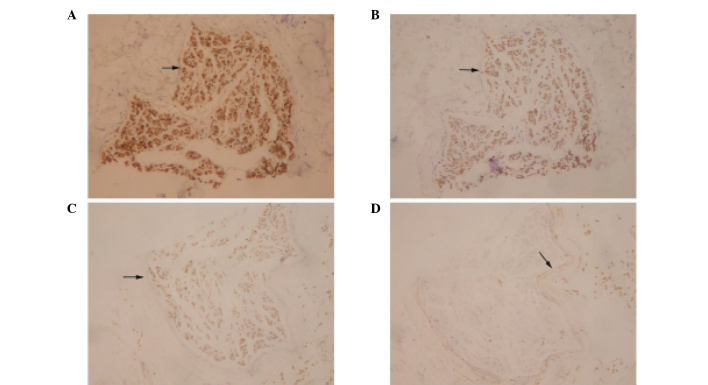
Immunohistochemical analyses of the sural nerve revealed (A) S-100 strongly positive immunoreactivity (arrow), (B) NSE moderately positive immunoreactivity (arrow; abundant in the cell bodies), (C) NF mildly positive immunoreactivity (arrow) and (D) EMA mildly positive immunoreactivity. NSE, neuron-specific enolase; NF, neurofilament; EMA, epithelial membrane antigen.

Immunohistochemical analysis (Fig. 5) demonstrated that the majority of the cells within the nerve fascicles were strongly positive for S-100, moderately positive for neuron-specific enolase (NSE) and mildly positive for neurofilament (NF), while the perineurium was mildly positive for epithelial membrane antigen (EMA). No anatomical abnormalities were observed and the density of the fibers moderately decreased.

Following intravenous immunoglobulin refusion treatment, the patient was administered one course of small-dose (20 mg/day) prednisone, but without any benefit. Next, the patient received a five-day course of intravenous immunoglobulin (400 mg/kg/day). The clinical symptoms, including numbness and ataxia, began to improve two weeks after admission. Follow-up examinations for 30 months revealed only generalized areflexia without ataxia.

## Discussion

Wartenberg (3) discussed the concept of a sensory equivalent to the ascending paralysis of GBS in 1958, while the study by Asbury (5) provided diagnostic criteria for a sensory loss and areflexia variant in 1981. However, whether their clinical and electrodiagnostic features are pathognomonic of acute sensory neuronopathy or sensory GBS remains controversial. To date, reported clinical cases meeting these criteria have been scarce, for which there are two main reasons. Firstly, these cases always demonstrate a degree of motor weakness or abnormalities in motor NCSs, which suggests that the cases are predominantly sensory GBS, rather than purely sensory GBS. Secondly, acute sensory neuropathy represents two clinical syndromes: Acute sensory neuronopathy involving the dorsal root ganglia and sensory GBS, an acute demyelinating neuropathy that presents clinically with involvement of the sensory peripheral nerve only. The total protein in the CSF is not useful for distinguishing sensory GBS from sensory neuronopathy since the protein level may be elevated in the two disorders.

Demonstrating electrophysiological evidence of sensory demyelination in GBS may be difficult, with the exception of usual presentation, where there is evidence of demyelination on motor NCSs (4). Sensory neuronopathy can be differentiated by the absent sensory nerve action potentials in the presence of normal motor conduction (6). In the present case, the regenerated nerve fibers were observed via light microscopy of the sural nerve specimen and the sagittal T2-weighted MRI scan of the cervical spine, which demonstrated a normal appearance of the posterior column.

Furthermore, immunohistochemical analysis of the sural nerve specimen may be useful in distinguishing sensory GBS from sensory neuronopathy (7). NF, NSE and S-100b proteins are nervous system-specific proteins (8), and EMA is a perineurial-specific protein. An increase in NF and NSE levels reflect axonal damages in radices, while that of S-100b indicates Schwann cell damage associated with demyelination. A number of studies have indicated that the majority of patients with normal NSE and S-100b levels demonstrate a good recovery, while markedly high levels of NSE are associated with long-term recovery and residual disability (9,10,11,12,13). An additional study demonstrated that high NF levels in the CSF, indicating proximal axonal damage, at disease onset are a robust predictor of poor motor recovery (14).

In the present study, immunohistochemical analysis revealed that the majority of cells within the nerve fascicles were strongly positive for S-100, moderately positive for NSE and mildly positive for NF, while the perineurium was mildly positive for EMA. Moderate positivity for NSE and mild positivity for NF indicated that the axonal damages were not in radices, while the strong positivity for S-100 indicated Schwann cell damage associated with demyelination. In addition, electron microscopy revealed normal myelinated and unmyelinated axons with Schwann cell nucleus breakdown, which confirmed the outcome of the immunohistochemical analyses. Furthermore, the perineurial cell proliferated and retained its EMA immunoreactivity within the dermal nerve sheath myxomas, Morton’s metatarsalgia and traumatic neuromas. It should be possible to immunohistochemically ‘dissect’ the structure of the peripheral nerve and its lesions by EMA and S-100, since EMA is used to indicate perineurial cells and S-100 to identify Schwann cells (15). In the present study, the perineurium revealed strong S-100 protein expression but mild EMA immunoreactivity, which may reflect normal perineurial structures or a mild lesion.

In conclusion, the aforementioned observations indicated that the present case was most likely pure sensory GBS, not sensory neuronopathy; thus, had a good prognosis. However, mild numbness and generalized areflexia symptoms remained with the patient. This is characteristic of GBS, which residual sensory deficit is present in a considerable number of patients and frequently has a disruptive effect, even several years following the onset of GBS (16).
